# Prenatal training module (PTM) with newborn simulation model to enhance primipara mother’s knowledge and skill on newborn care in Lower-Middle-Income Setting: A quasi-experimental study

**DOI:** 10.12688/f1000research.177800.1

**Published:** 2026-04-18

**Authors:** Kavya C, Shashidhara YN, Manjula U

**Affiliations:** 1 Department of Community Health Nursing, Manipal College of Nursing, Manipal Academy of Higher Education, Manipal, Karnataka, 576104, India

**Keywords:** Breast Feeding/methods; Maternal Knowledge; Infant Care/standards; Hygiene/standards; Health Education/methods

## Abstract

**Background:**

Parental knowledge of newborn care is essential, as it can influence the newborn’s health, growth, and development. It is challenging due to a lack of mothers’ knowledge and skills, insufficient resources, and limited training facilities. The study evaluates the effectiveness of the prenatal training module (PTM) on maternal knowledge and skills on newborn care.

**Methods:**

A quasi-experimental research design was adopted. Forty women at or beyond 36 weeks of gestation were recruited using a non-probability purposive sampling. The pre-test knowledge was assessed during the antenatal period for both the interventional and control groups. The interventional group received two sessions of prenatal training using a simulation, with a one-week interval. The post-test was conducted for both the Interventional and control groups on the third day of child birth.

**Results:**

There was an evident variation between post-test knowledge score (Z = -5.345; P < 0.05) and post-test skill score (Z = -5.144; P < 0.05) among mothers on newborn care between groups.

**Conclusions:**

Post-training skill demonstration and knowledge were markedly better among women in the interventional group than in the control group. The PTM with the newborn simulation model has shown success in improving knowledge and skills among mothers.

## Introduction

Newborn care is multidimensional and requires a wide range of practices and skills to ensure the newborn’s growth, development, health, and comfort, as newborns experience extensive transformations and multifaceted coordination within vital organs to survive after birth. ^1– 3^ Significant changes observed in cardiovascular, respiratory, endocrine, and metabolic systems. ^1, 3^ Newborn care is a critical aspect encompassing essential tasks to support physiological adjustment such as maintaining a stable temperature, airway and circulation management, breastfeeding, skin care, umbilical cord care, eye care, immunization, and are initiated soon after the newborn’s birth. ^4, 5^ Furthermore, Newborns are born with underdeveloped systems, including the integumentary, gastrointestinal, musculoskeletal, and renal systems, making them highly susceptible to multiple complications. ^1^ Adequate newborn care is crucial to reduce the respiratory distress, infections, morbidity, and mortality, thereby improving survival rates. ^1^ Newborn care practices play a key role in global neonatal mortality reduction, predominantly in low and middle-income countries (LMICs). ^6^

The Neonatal Mortality Rate (NMR) remains at 36 deaths per 1,000 live births globally, which is contributing to approximately 5.1 million neonatal deaths annually. Reports show that 98 percent of all neonatal fatalities occurred in developing countries. ^7, 8^ In India, the prevalence of early neonatal mortality is 2.1% noted in the year 2019–2021. ^5^ Mothers must receive hands-on training and education on newborn care, ideally through simulation models that mimic real-life scenarios. ^9^ This fosters mother’s knowledge and skill, which enables mothers to play a crucial role in preventing complications, promoting adaptation, and ultimately saving lives. ^9^ Moreover, empowering mothers with the necessary skills and knowledge can also have a positive impact on the overall healthcare system, improving the quality of care provided. ^10^

Healthcare specialists play a vital role in educating communities, families, and individuals on routine newborn care, thereby promoting healthier outcomes and preventing predominant health concerns. The challenges in Lower-Middle-Income settings are greatly exacerbated by a critical shortage of resources in learning and severely limited access to training centres for primipara mothers. This glaring lack of resources and expertise has detrimental consequences, chiefly concerning morbidity and mortality rates. India’s ambition is to achieve the Sustainable Development Goal (SDG) 3 to bring down the newborn mortality rate to 12 or less per 1000 births by 2030. This study attempts to address a major problem by training mothers in rural areas of Karnataka, India, on newborn care through a Prenatal Training Module (PTM) and simulation model, with the goal of augmenting their knowledge and skills in this area. Thereby, this study aims to decrease mortality and morbidity among newborns, ultimately resulting in better health outcomes and a healthier population. Therefore, the study aimed to determine the effectiveness of the prenatal training module on newborn care knowledge and skills among mothers. The primary outcomes were post-test knowledge and skill scores among mothers in the interventional and control groups.

## Materials and methods

### Research design

A quasi-experimental study was undertaken to assess the effectiveness of PTM with a newborn simulation model on the knowledge and skills among primipara mothers on newborn care in the year 2017–2018. Two distinct hospitals are chosen from the low- to middle-income setting of the Udupi district, Karnataka, India, to prevent cross-group data contamination; one hospital was allocated purposively to the experimental group and the other to the control group. The study employed a pre-test post-test comparison group design to measure the knowledge variable. Conversely, a post-test only control group design was used to assess the skill variable. This design choice is crucial to prevent biased outcomes, as comparing skill levels on a simulation model to those of a newborn would be fundamentally inaccurate.

### Selection and description of participants

The study outlined that to recruit primipara mothers with a gestational age of 36 weeks or more. Participants had to meet the following criteria to be eligible for inclusion: willingness and availability at the time of data collection, and the ability to speak, read, and write in Kannada. Conversely, high-risk pregnancies characterized by threatened abortion, gestational hypertension, gestational diabetes, placenta previa, and abruptio placentae were excluded. The sample size was estimated using the comparison of means formula, a method derived from a previous study conducted by Tyseer & Hanan, 2015. ^11^

$$n=\frac{2{\left(Z_{\alpha /2}+Z_{\beta }\right)}^{2}\sigma^{2}}{d^{2}}$$

Where,

n = Minimum sample size required.

Z 1-α/2 = 1.96.

Zβ = 0.84 (Power).

σ = Standard deviation.

d = Clinically significant difference.

10% attrition.

The sample size for each group was arbitrarily determined to be 17, yet a total of 20 subjects were included in each group. Eligible participants were carefully chosen through a non-probability sampling method, specifically purposive sampling, at the selected hospitals within the Udupi district of India.

### Tools and data collection instruments:

The demographic tool includes baseline information related to antenatal mothers and consists of 12 questions. The knowledge questionnaire on newborn care comprises 32 questions, covering topics such as thermoregulation, breastfeeding, and personal hygiene. The newborn care skill checklist includes 28 items, also focusing on thermoregulation, breastfeeding, and personal hygiene.

The tools were validated by seven subject experts and found to be valid. The reliability of the knowledge questionnaire was assessed using the split-half technique and Spearman’s prophecy formula, yielding a reliability coefficient of r = 0.812. The reliability of the skill checklist was determined using the inter-rater method, with a coefficient of r = 0.9.

### Description of the intervention: Prenatal training module on Newborn Care

The prenatal training module was validated, and expert suggestions were incorporated. The module includes information on normal physiology, purpose, procedure, risks and benefits, and abnormalities related to thermoregulation, breastfeeding, and personal hygiene. Tools and modules were developed in accordance with WHO and UNICEF guidelines. ^12– 14^ This training media was used to educate antenatal mothers on thermoregulation, breastfeeding, and personal hygiene of newborns using the prenatal training module and newborn simulation model. The training was delivered in small groups of less than five antenatal mothers in a selected hospital by the researcher for one hour, and the second training session was held after a week. There is no risk or harm associated with the training.

### Recruitment and data collection procedure

**Figure 1.**
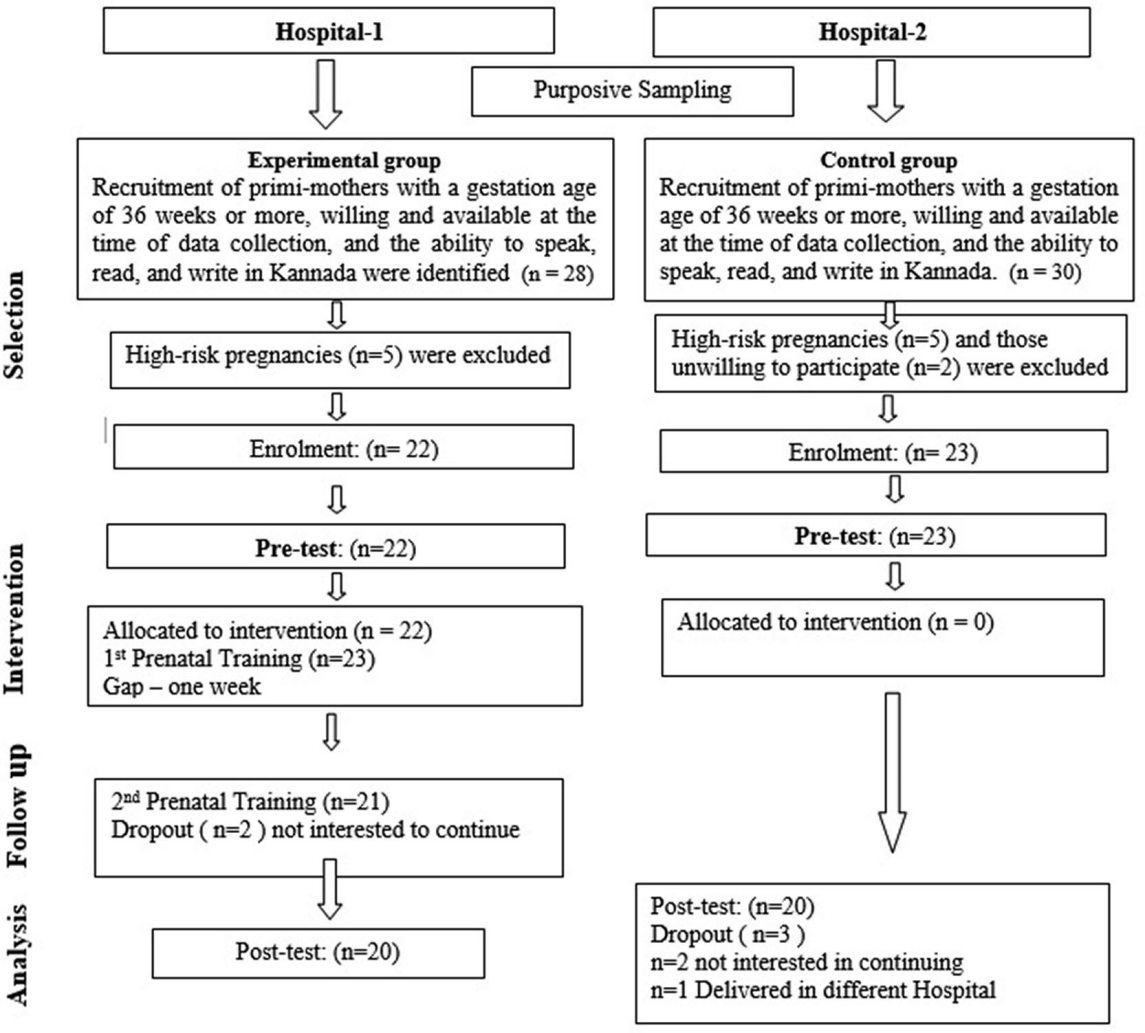
Participant flow.

Two distinct hospitals were chosen from the Udupi district, Karnataka, India, to prevent cross-group data contamination; one hospital was allocated purposively to the experimental group and the other to the control group. Participants who visited selected hospitals for routine antenatal care services are screened for eligibility and willingness. Further, the participation information sheet was explained, and written consent was obtained from eligible participants from December 2017 to April 2018. A structured, validated, pretested, and reliable knowledge questionnaire and observational checklist were developed based on recommendations. During the first visit, initial baseline data collection and pre-test knowledge assessment were conducted using baseline data on antenatal mothers and a structured knowledge questionnaire on newborn care. This assessment occurred at a gestational age of 36 weeks or more in both groups. In the interventional group, two prenatal training sessions were held, with a seven-day interval between them, during the antenatal period. The intervention used in the study was video-recorded prenatal training with a newborn simulation model, focusing on newborn care. This training media was used to educate antenatal mothers on thermoregulation, breastfeeding, and personal hygiene of newborns using the prenatal training module and newborn simulation model. The training was delivered in small groups of less than five antenatal mothers in a selected hospital by the researcher for one hour. Post-test knowledge and skill assessment were conducted on the third day of birth from both groups using a standardized questionnaire to assess knowledge and an observational tool to evaluate skill. The participant flow is depicted in Figure 1. Both groups continued to receive routine antenatal care services during the study period. No unintended events or harms were reported during the study.

### Data analysis

All mothers who completed both the pre-test and post-test assessments were included in the final analysis. The data were carefully coded and thoroughly documented in a master data sheet, and a comprehensive analysis was carried out using SPSS version 16. Descriptive statistics, including Percentage, mean, Standard deviation, and frequency. Inferential statistics, specifically the Mann-Whitney U-test and the Wilcoxon signed-rank test, were used to examine the data, as the data was not normally distributed. No missing data were identified, and no subgroup analysis was conducted.

**Ethical Considerations:** The Administrative permission and Institutional ethical committee approval were obtained from Kasturba Medical College and Kasturba Hospital (IEC No: IEC 743/2017). Additionally, Clinical Trials Registry- India’s approval was obtained (CTRI registration number - CTRI/2017/12/011013). Registered on: 28/12/2017. Informed consent was obtained from the study participants before the data collection, and their confidentiality and anonymity were maintained.

## Results

### Description of participants’ characteristics

The results showed that most respondents, with a mean age of 26.10 ± 2.5, were in the interventional group, and a mean age of 26.20 ± 4.2 was observed in the control group. The respondents in the interventional group, i.e., seven (35%) and ten (50%) in the control group, had a minimum qualification of graduation. The majority of the interventional group had a family income of ₹ 10,001-20,000, whereas the income was ≤10,000 in the control group. The majority of respondents belonged to a nuclear family in both groups. In the interventional group, 60 percent of the participants, who were 12 individuals, fell into this category. Similarly, in the control group, 75 percent of the participants, or 15 individuals, were part of a nuclear family.

### Description of the knowledge scores of newborn care among mothers

**Figure 2.**
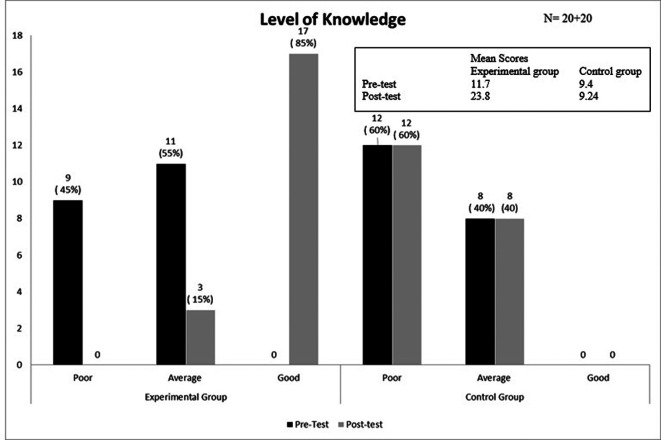
Bar diagram presenting the pre- and post-test levels of knowledge on newborn care among mothers in each group.

The area-wise distribution of knowledge scores in the interventional and control groups is shown in Tables 1– 3, which indicates that the interventional group showed an increase in post-test mean scores, whereas the control group did not. The results suggest that the intervention, PTM, had a positive influence on the mother’s knowledge on newborn care in the interventional group, as evidenced by an increase in their knowledge score. On the other hand, this improvement was not observed in the control group, indicating that the PTM intervention was the key factor behind the gain in knowledge. The level of knowledge score in pre- and post-test among mothers in the group is presented in Figure 2.

**Table 1.** Area-wise distribution of knowledge scores in the interventional and control groups N = 20 + 20 = 40.

ITEMS	Interventional Group				Control Group			
	Pre-test		Post test		Pre-test		Post test	
	Correct f(%)	Mean ± SD	Correct f(%)	Mean ± SD	Correct f(%)	Mean ± SD	Correct f(%)	Mean ± SD
**Thermoregulation Domain**								
Thermoregulation meaning	9(45%)	.45 **±** **.**51	15(75%)	.75 ± .44	4 (20%)	.20 ± .41	5 (25%)	.25 ± .44
Normal body temperature	1 (5%)	.05 ± .22	9(45%)	.45 ± .51	2 (10%)	.10 ± .31	3(15%)	.15 ± .37
Risk factors	2 (10%)	.10 ± .31	16(80%)	.80 ± .41	3(15%)	.15 ± .37	2 (10%)	.10 ± .31
Cause of low body temperature	7 (35%)	.35 ± .49	9 (45%)	.45 ± .51	2 (10%)	.10 ± .31	2 (10%)	.10 ± .31
Types of heat loss	6(30%)	.30 ± .47	10(50%)	.50 ± .51	6(30%)	.30 ± .47	7 (35%)	.35 ± .49
Thermoregulation Techniques	9 (45%)	.45 ± .51	16(80%)	.80 ± .41	6(30%)	.30 ± .47	5 (25%)	.25 ± .44
Signs of low body temperature	2 (10%)	.10 ± .31	19(95%)	.95 ± .22	2 (10%)	.10 ± .31	2 (10%)	.10 ± .31
Measures to achieve warmth	10(50%)	.50 ± .51	13(65%)	.65 ± .49	2 (10%)	.10 ± .31	2 (10%)	.10 ± .31
**Thermoregulation Overall**		**2.30 ± 1.34**		**5.35 ± .933**		**1.4 ± 1.03**		**1.40 ± .99**

**Table 2.** Area-wise distribution of knowledge scores in the interventional and control groups N = 20 + 20 = 40.

Items	Interventional Group				Control Group			
	Pre-test		Post test		Pre-test		Post test	
	Correct f(%)	Mean ± SD	Correct f(%)	Mean ± SD	Correct f(%)	Mean ± SD	Correct f(%)	Mean ± SD
**Breastfeeding Domain**								
Breastfeeding Benefits: Newborn	10(50%)	.50 ± .51	11(55%)	.55 ± .51	3(15%)	.15 ± .37	2 (10%)	.10 ± .31
Breastfeeding Benefits: Mother	8 (40%)	.40 ± .50	11 55%)	.55 ± .51	7 (35%)	.35 ± .49	7 (35%)	.35 ± .49
Ideal time for first breastfeeding	8(40%)	40 ± .50	18(90%)	.90 ± .31	5 (25%)	.25 ± .44	5 (25%)	.25 ± .44
Frequency of breastfeeding	5 (25%)	.25 ± .44	18(90%)	.90 ± .31	4 (20%)	.20 ± .41	3(15%)	.15 ± .37
Duration of each feed	10(50%)	.50 ± .51	15(75%)	.75 ± .44	4 (20%)	.20 ± .41	4 (20%)	.20 ± .41
Exclusive breastfeeding duration	10(50%)	.50 ± .51	18(90%)	.90 ± .31	14 (70%)	.70 ± .47	14 (70%)	.70 ± .47
Breast holding technique	5 (25%)	.25 ± .44	15(75%)	.75 ± .44	3(15%)	.15 ± .37	4 (20%)	.20 ± .41
Ideal maternal position	9(45%)	.45 ± .51	17(85%)	.85 ± .37	5 (25%)	.25 ± .44	5 (25%)	.25 ± .44
Newborn holding technique	2(10%)	.10 ± .31	13(65%)	.65 ± .49	2 (10%)	.10 **±** .31	2 (10%)	.10 ± .31
proper latch technique	11 (55%)	.55 ± .51	20(100%)	1.00 ± .00	15(75%)	.75 ± .44	15(75%)	.75 ± .44
Burping position	1 (5%)	.05 ± .22	7 (35%)	.35 ± .49	2 (10%)	.10 ± .31	1 (5%)	.05 ± .22
Breastfeeding myths versus reality	15 (75%)	.75 ± .44	17(85%)	.85 ± .37	14 (70%)	.70 ± .47	14 (70%)	.70 ± .47
Improper breastfeeding technique complication: mother	8 (40%)	.40 ± .50	12(60%)	.60 ± .50	2 (10%)	.10 ± .31	1 (5%)	.05 ± .22
Improper breastfeeding technique complication: baby	13(65%)	.65 ± .49	17(85%)	.85 ± .37	11 55%)	.55 ± .51	9(45%)	.45 **±** .51
Ways to enhance milk production	10(50%)	.50 ± .51	16(80%)	.80 ± .41	10(50%)	.50 ± .51	9(45%)	.45 ± .51
**Breastfeeding Overall**		**6.25 ± 2.19**		**11.25 ± 2.26**		**5.05 ± 2.35**		**4.75 ± 2.19**

**Table 3.** Area-wise distribution of knowledge scores in the interventional and control groups N = 20 + 20 = 40.

Items	Interventional Group				Control Group			
	Pre-test		Post test		Pre-test		Post test	
	Correct f(%)	Mean ± SD	Correct f(%)	Mean ± SD	Correct f(%)	Mean ± SD	Correct f(%)	Mean ± SD
**Personal hygiene Domain**								
Purpose of diaper care functions of skin	13(65%)	.65 ± .49	18(90%)	.90 ± .31	12(60%)	.60 ± .50	13(65%)	.65 ± .49
Care of skin	5 (25%)	.25 ± .44	7(35%)	.35 ± .49	4 (20%)	.20 ± .41	4 (20%)	.20 ± .41
diaper care measures	9(45%)	.45 **±** .51	17(85%)	.85 ± .37	9(45%)	.45 ± .51	9(45%)	.45 ± .51
water temperature checking ways	1 (5%)	.05 ± .22	12(60%)	.60 ± .50	4 (20%)	.20 ± .41	4 (20%)	.20 ± .41
Diaper care helps to observe	5 (25%)	.25 ± .44	18(90%)	.90 ± .31	5 (25%)	.25 ± .44	4 (20%)	.20 ± .41
Umbilical cord care	6(30%)	30 ± .47	17(85%)	.85 ± .37	5 (25%)	.25 ± .44	6(30%)	.30 ± .47
Proper direction to clean eyes	14 (70%)	.70 ± .47	19(95%)	.95 ± .22	11 55%)	.55 ± .51	12(60%)	.60 ± .50
Directions for cleaning the	4 (20%)	.20 ± .41	19(95%)	.95 ± .22	7 (35%)	.35 ± .49	7 (35%)	.35 ± .49
perineum of a newborn	6(30%)	.30 ± .47	17(85%)	.85 ± .37	3(15%)	.15 ± .37	3(15%)	.15 ± .37
**Personal hygiene Overall**		**3.15 ± 1.69**		**7.20 ± 1.336**		**3 ± 1.65**		**3.10 ± 1.59**
**Overall**		**11.70** ± **3.59**		**23.80** ± 3.60		**9.40 ± 3.60**		**9.25 ± 3.45**

### Description of the skill scores of newborn care among mothers

**Figure 3.**
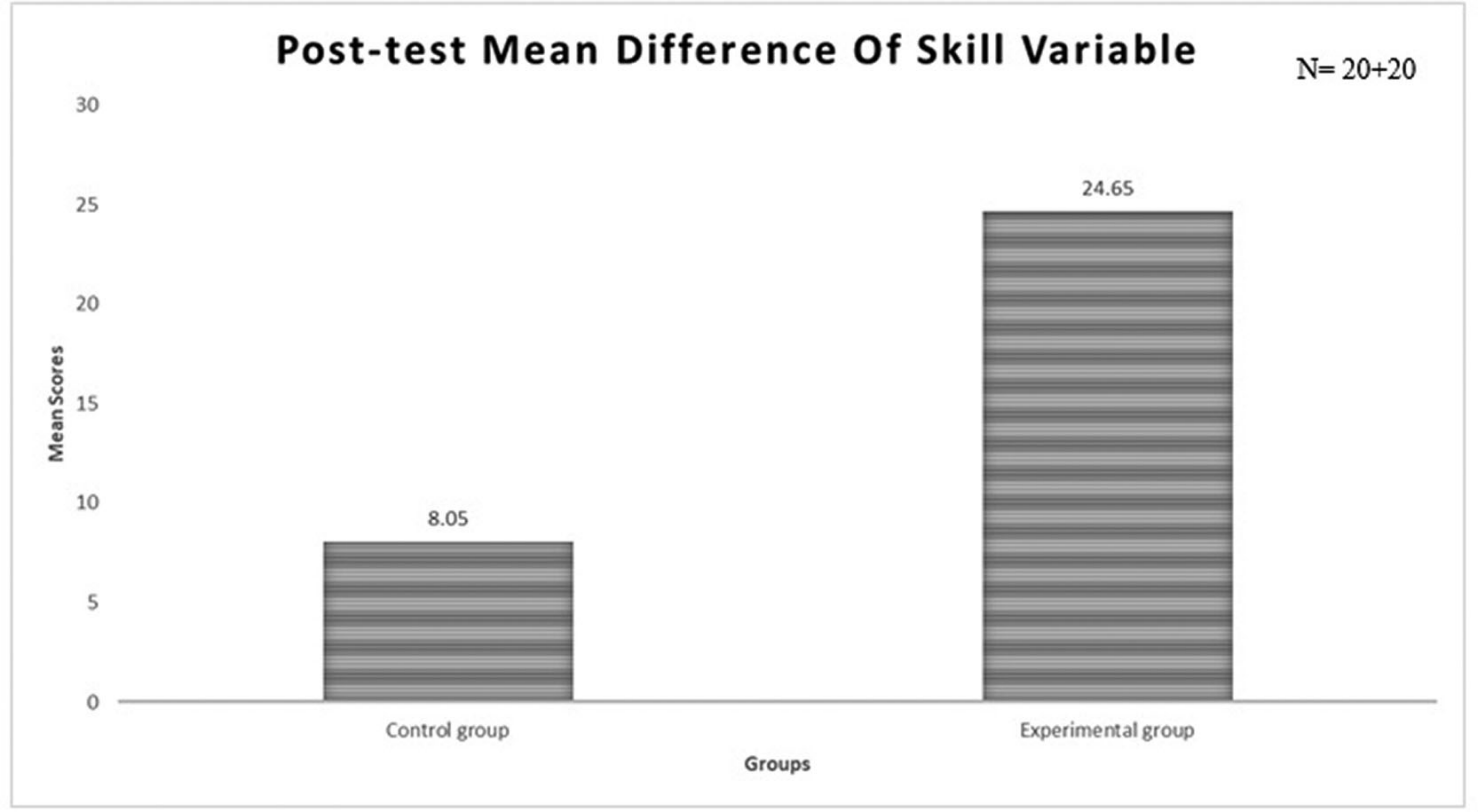
Bar diagram presenting post-test mean scores of skills on newborn care among mothers in each group.

The area-wise distribution of skill scores in the interventional and control groups and the post-test mean score are presented in Tables 4– 8 and Figure 3, respectively. The results reveal that the overall post-test mean score in the interventional group was notably higher than that in the control group. Consequently, it can be inferred that participation in PTM contributed to an improvement in skill on newborn care among mothers who participated in the intervention group, as evidenced by a gain in skill scores. In contrast, the control group failed to demonstrate any improvement in skill scores, as they did not participate in prenatal training on newborn care.

**Table 4.** Area-wise distribution of skill scores in the interventional and control groups N = 20 + 20 = 40.

Skill	Interventional group					Control group				
	Yes		No		Remarks(Only for no response)	Yes		No		Remarks(Only for no response)
	F	%	F	%		f	%	F	%	
**Thermoregulation Domain**										
Warm hands	9	45	11	55	Touched the baby without warming hands	0	0	20	100	Touched baby without warming hands
Place the cloth on firm surface	19	95	1	5	Places baby on the lap	19	95	1	5	Places baby on the lap
Fold a little from one corner	20	100	0	0	NA	12	60	8	40	Did not fold from the corner
Position the newborn such that the newborn’s head is on the folded corner	20	100	0	0	NA	17	85	3	15	Placed the baby on the edge of the square clothes
Wrap the head and then wrap the cloth over one side of the newborn’s chest	19	95	1	5	Did not cover the baby’s head	17	85	3	15	Did not cover the baby’s head
Repeat for the other side	19	95	1	5	Leaves baby’s chest open	13	65	7	35	Leaves chest open
Secure the feet by tucking the lower corner over them	18	90	2	10	Leaves baby’s leg open	6	30	14	70	Leaves baby’s leg open
**Thermoregulation Overall**	**Mean ± SD = 6.35 ± 0.49**					**Overall Mean ± SD = 4.2 ± 1.54**				

**Table 5.** Area-wise distribution of skill scores in the interventional and control groups N = 20 + 20 = 40.

Skill	Interventional group					Control group				
	Yes		No		Remarks (Only for no response)	Yes		No		Remarks (Only for no response)
	f	%	f	%		f	%	F	%	
**Breastfeeding –position Position the newborn for feeding**										
Sit straight and use a pillow to support her back	18	90	2	10	Bending forward	6	30	14	70	Bending forward
Clean the breast with a warm water-soaked cloth	9	40	11	55	Breastfeeding without washing the breast	1	5	19	95	Breastfeeding without washing the breast
Use a pillow under the newborn	16	80	4	20	Baby on lap (more gaps between the baby’s chest and the mother)	7	35	13	65	Baby on lap (more gaps between the baby’s chest and the mother)
Rest the newborn’s head on her elbow joint, and rest the newborn’s body on her forearm	18	90	2	10	Holding a baby’s head with the palm	9	45	11	55	Holding a baby’s head with the palm
The newborn’s chest should touch the mother’s chest	15	75	5	25	more gaps between the baby’s and the mother’s chest	2	10	18	90	more gaps between the baby’s chest and the mother’s

**Table 6.** Area-wise distribution of skill scores in the interventional and control groups N = 20 + 20 = 40.

Skill	Interventional group					Control group				
	Yes		No		Remarks (Only for no response)	Yes		No		Remarks(Only for no response)
	f	%	f	%		f	%	F	%	
**Position to hold breast:** ‘C’ position: Grasp	17	85	3	15	Scissor hold	2	10	18	90	Scissor hold
**Latch**										
Sit straight	18	90	2	10	Bending forward	6	30	14	70	Bending forward
Use a pillow under the newborn	16	80	4	20	Baby on lap (more gaps between the baby’s chest and the mother)	7	35	13	65	Baby on lap (more gaps between the baby’s chest and the mother)
The newborn’s chest should face the mother’s chest	20	100	0	0	more gaps between the baby’s chest and the mother’s	8	40	12	60	more gaps between the baby’s chest and the mother’s
Insert the breast inside the newborn’s mouth when the mouth is wide open	19	95	1	5	Did not wait till the baby opens its mouth widely	8	40	12	60	Did not wait till the baby opens its mouth widely
Make sure that most of the black part of the breast is inside the newborn’s mouth	20	100	0	0	Baby sucking only the nipple	0	0	20	100	Baby sucking only the nipple
Place your little finger inside the newborn’s mouth after feeding the newborn, to prevent nipple pulling	13	65	7	35	Pulling the nipple from the baby’s mouth	0	0	20	100	Pulling of the nipple from the baby’s mouth

**Table 7.** Area-wise distribution of skill scores in the interventional and control groups N = 20 + 20 = 40.

Skill	Interventional group					Control group				
	Yes		No		Remarks (Only for no response)	Yes		No		Remarks (Only for no response)
	F	%	F	%		f	%	f	%	
**Position the newborn for burping**										
Place the newborn on the shoulder	20	100	0	0	NA	20	100	0	0	NA
Rub or tap the newborn’s back from the bottom to the top	15	75	5	25	Taping on the back without following any direction	0	0	20	100	Taping on the back without following any direction	
**Breastfeeding Overall**	**Mean ± SD = 11.70 ± 1.29**					**Overall Mean ± SD = 3.85 ± 1.69**				

**Table 8.** Area-wise distribution of skill scores in the interventional and control groups N = 20 + 20 = 40.

Skill		Interventional group				Control group					
	Yes		No		Remarks (Only for no response)	Yes		No		Remarks (Only for no response)
	f	%	f	%		f	%	f	%	
**Personal hygiene- Diaper care**										
Take a square cloth.	19	95	1	5	Used a disposable diaper	0	0	20	100	Used a disposable diaper
Place one corner of the square cloth so that it faces the mother.	19	95	1	5	Used a disposable diaper	0	0	20	100	Used a disposable diaper
Fold the left corner approximately to the centre, then fold the right corner to the centre.	17	85	3	15	Folded cloth, such as a triangular shape	0	0	20	100	Used a disposable diaper
Fold the upper triangular corner over the previous folds.	16	80	4	20	Skipped this step	0	0	20	100	Used a disposable diaper
Fold the bottom corner, fold it front again.	16	80	4	20	Skipped this step	0	0	20	100	Used a disposable diaper
Place the newborn on the cloth diaper prepared.	19	95	1	5	Used a disposable diaper	0	0	20	100	Used a disposable diaper
Fold the bottom corner of newborn and wrap the side corners around the waist. Secure it.	19	95	1	5	Used a disposable diaper	0	0	20	100	Used a disposable diaper
**Personal hygiene Overall**	**Mean ± SD = 6.60 ± 9.9**									
**Total**	**Mean ± SD = 24.65 ± 1.56**					**Mean ± SD = 8.05 ± 2.35**				

### Thermoregulation domain

About 9(45%) participants warmed their hands in the interventional group, whereas no one performed in the control group. Participants secured the feet by tucking the lower corner in the interventional group 18 (90%), whereas 6 (30%) participants performed in the control group. The area-wise distribution of skill scores in the interventional and control groups is depicted in Table 4.

### Breastfeeding domain

In the present study, 18(90%) participants sat straight and used the pillow to support their back in the interventional group, whereas 6 (30%) participants performed in the control group. 9(40%) of participants used the pillow under the newborn in the interventional group, whereas 7(35%) of participants performed in the control group. About 18(90%) participants placed the newborn’s head on their elbow joint and rested the newborn’s body on the forearm in the interventional group, whereas 9(45%) participants performed in the control group. Most of the participants 15(75%) placed the newborn’s chest in a way that it touched the mother’s chest in the interventional group, whereas 2(10%) participants performed in the control group. Mother used the ‘C’ Grasp in the interventional group 17(85%), whereas 2(10%) performed in the control group. The area-wise distribution of skill scores in the interventional and control groups is depicted in Tables 5– 7.

### Personal hygiene domain

Most of the participants took a square cloth to prepare a cloth diaper in the interventional group 19(95%), whereas none performed in the control group; instead, they used the disposable diapers. The area-wise distribution of skill scores in the interventional and control groups is depicted in Table 8.

The data show that the majority of participants followed most of the steps of newborn care in the interventional group compared to the control group, as presented in Tables 4– 8.

### Effectiveness of PTM

The data presented in Table 9 show that there is statistically significant variation in post-test knowledge scores on newborn care between groups (Z = −5.345; P < 0.05). Hence, it was inferred that PTM is effective in improving mothers’ knowledge on newborn care in the interventional group. This implies that the interventional group had a significantly higher level of knowledge compared to the control group.

**Table 9.** Mann-Whitney U-test computed between post-test knowledge score between interventional and control groups N = 20 + 20.

Knowledge	Median	IQR (Q _1_, Q _3_)	Z-value	P-value
Interventional	25.00	22–26	-5.345	0.001
Control	10	6.25–11		

P < 0.05.

The data presented in Table 10 demonstrated that there is a statistically significant difference between the post-test skill score on newborn care among the mothers among groups (Z = −5.144; P < 0.05). Findings indicate that the interventional group exhibited a notable improvement in skills, thereby underscoring the effectiveness of PTM in enhancing skills among the interventional group. This indicates that the interventional group had a higher level of proficiency in newborn care compared to the control group.

**Table 10.** Mann-Whitney U-test computed between post-test skill score between interventional and control groups N = 20 + 20 = 40.

Skill	Median	IQR (Q _1_– Q _3_)	Z-value	P-value
Interventional	25	23–26	-5.144	0.001
Control	8.5	6–10		

P < 0.05.

## Discussion

In the present study, nine (45%) of the subjects in the interventional group had poor knowledge, while 11 (55%) had average knowledge in the pre-test. In contrast, the control group consisted of 12 (60%) subjects with poor knowledge and eight (40%) with average knowledge. Following the intervention, the interventional group comprised 17 (85%) subjects with good knowledge and three (15%) with average knowledge, whereas the control group remained at 12 (60%) with poor knowledge and eight (40%) with average knowledge.

The findings presented in Figure 2 are substantiated by the results of a descriptive survey carried out to determine the practice and knowledge of personal hygiene and newborn care in rural areas at Thiruvallur, Tamil Nadu. The data revealed a concerning trend, with 70 percent of mothers exhibiting inadequate knowledge. Furthermore, 30 percent of participants demonstrated moderately adequate knowledge; a substantial portion of the population remains in need of education. Notably, none of the participants possessed adequate knowledge. The survey also highlighted the disparity between knowledge and practice, with 63.3 percent of participants exhibiting poor personal hygiene practices. Conversely, 36.7 percent of participants demonstrated satisfactory practice. However, the absence of participants with good practice underscores the need for a comprehensive teaching program to address the knowledge and practice gaps. ^15^

In the present study, the results are well supported by the findings of a study conducted to determine the mother’s knowledge and practice on breastfeeding in the primary healthcare unit at Ismailia, Egypt. The results revealed that 64 percent of mothers employed correct breastfeeding positions, whereas 33 percent failed to adopt the recommended positions. Furthermore, 61 percent of mothers were unaware of the benefits associated with breastfeeding, and 25.5 percent possessed knowledge of the correct procedure of breastfeeding. Consequently, the researcher concluded that mothers exhibit poor knowledge and practice of breastfeeding. ^16^

Post-implementation of the PTM, the results of the present study show noticeable improvement in the mother’s knowledge and skills on newborn care. The statistical analysis reveals a highly significant difference in the knowledge (Z = −5.345; P < 0.001) and skill (Z = −5.144; P < 0.001) of mothers in the interventional group.

The results presented in Tables 9 and 10 are consistent with the findings of a study conducted to appraise a structured educational program on mother’s knowledge on neonatal hypothermia at hospitals in Belgaum, Karnataka. The study focused on assessing the impact of PTP on postnatal mothers’ understanding of neonatal hypothermia prevention and management. The findings indicated that it was effective in optimizing knowledge, with a statistically significant outcome (t = 15.6; p < 0.05). This outcome suggests that the structured educational program was successful in enhancing the mothers’ knowledge and awareness about neonatal hypothermia. ^17^

The results presented in Tables 9 and 10 are in agreement with findings of a study designed to evaluate video-supported essential newborn care teaching strategy on knowledge among mothers in a selected ward of hospitals in Vellore, Tamil Nadu. The findings indicated that teaching was efficient in boosting mother’s knowledge, as evidenced by a marked gain in mean knowledge score from 9.0 to 34.46 (p < 0.001). ^18^

The results presented in Table 2 are reinforced by a study aimed at evaluating an awareness program in enhancing breastfeeding knowledge among ASHA workers in Udupi district, Karnataka. The findings indicated a noteworthy improvement in knowledge scores, with a mean rise from 13.6 to 17.4 (p < 0.001) following the post-test assessment. ^19^

The results presented in Tables 9 and 10 are consistent with the findings of a study examining the impact of video-based teaching on breastfeeding practices and knowledge among 60 mothers at Wardha, Maharashtra. The findings indicated a substantial advancement in post-test knowledge (t = 8.528; p < 0.05) and post-test practice (t = 6.281; p < 0.05). ^20^ Although the data were collected in 2017, a review of the current evidence-based guidelines (2025) indicates that no major revision to newborn care practices has occurred during this period. Therefore, the original module content remained valid and relevant. Moreover, Newborn mortality and morbidity are an ongoing public health priority aligned with SDG goal 3.2. Thus, the modules continue to be appropriate for educational use. ^12– 14^

### Limitations of the study

The researchers believe that the present study yielded useful results; it is important to accept the limitations.

• The skills of the mothers on newborn care were observed by the investigator at one point of time.
• The time gap between the 2nd training session and post-test could not be fixed as the labour was unpredictable after the 36th week of gestation.
• Post-test on bathing skills of mothers was not assessed as newborn bathing is encouraged after the cord falls at home (as per hospital policies).
• There are differences in the hospital policy. Hence, mothers were selected from different hospitals for the control and intervention groups.
• The generalizability may be limited as two hospitals from a specific geographic region, and due to the utilization of a non-randomized study design.

## Conclusion

The primary caregiver, the mother, plays a key role in fulfilling the needs of a newborn. The effectiveness of newborn care is deeply reliant on the knowledge and skills of the mother, family, and society, which raises concerns about the potential for inadequate care. Research has revealed that mothers who received prenatal training demonstrated better knowledge and skills in newborn care compared to those who did not receive training. Moreover, the effectiveness of prenatal training has been steadily reflected through study findings, underscoring its value in promoting effective newborn care. As a result, healthcare professionals can rely on this module to educate mothers on newborn care, thereby promoting effective care and reducing the risk of health problems, in line with the SDGs 3 and 4.

## Ethical considerations

Administrative permission and Institutional ethical committee approval were acquired from Kasturba Medical College and Kasturba Hospital (IEC No: IEC 743/2017). Additionally, approval was obtained from the Clinical Trials Registry- India (CTRI registration number - CTRI/2017/12/011013) Registered on: 28/12/2017. The study protocol and statistical analysis plan can be accessed on the Clinical Trials Registry of India.

## Disclaimers

The study is conducted for the partial fulfillment of the requirements for the degree of M.Sc. Nursing in Community Health Nursing and a dissertation submitted to Manipal Academy of Higher Education, Manipal. The Prenatal Training Module on Newborn Care has been applied for copyright under the Government of India through Manipal Academy of Higher Education (Diary Number: LD-43633/2025- CO).

## Data Availability

Zenodo: Newborn Care Knowledge and skill: Pre and Post-test Scores between groups. https://doi.org/10.5281/zenodo.18627020. ^21^ The project contains a coded dataset of pre- and post-test scores. Data are available under the terms of the Creative Commons Attribution 4.0 International license (CC-BY 4.0) and Creative Commons Zero v1.0 Universal. Zenodo: Primipara mother’s knowledge and skill on newborn care. https://doi.org/10.5281/zenodo.18955305. ^22^ The project contains the Consort Checklist, Outline of Teaching plan, Questionnaire, and Figures. Data are available under the terms of the Creative Commons Attribution 4.0 International license (CC-BY 4.0) and Creative Commons Zero v1.0 Universal.
