# Supplementary material for: Prenatal training module (PTM) with newborn simulation model to enhance primipara mother’s knowledge and skill on newborn care in Lower-Middle-Income Setting: A quasi-experimental study
Source: F1000Res. 2026 Apr 18;15:568. [Version 1] doi: 10.12688/f1000research.177800.1 (PMC13139849; doi:10.12688/f1000research.177800.1)
Supplement: Peer review history

# Reviewer report: Reviewer response for version 1

**DOI:** 10.5256/f1000research.196088.r479537

**Published:** 2026-05-05

**Authors:** Ankita jain

**Affiliations:** 1 Teerthanker Mahaveer Dental College and Research Centre, Moradabad, Uttar Pradesh, India

**Summary of the Article**

 This study evaluates the effectiveness of a prenatal training module (PTM) using a newborn simulation model in improving knowledge and skills related to newborn care among primipara mothers. A quasi-experimental design was used, with participants divided into intervention and control groups. Knowledge was assessed using a structured questionnaire during the antenatal period and after delivery, while skills were assessed postnatally. The study reports a statistically significant improvement in knowledge and skills in the intervention group compared to the control group.

 The topic is relevant and important in the context of maternal and child health, especially in lower-middle-income settings. However, several methodological and reporting issues need clarification and improvement.

**Major Comments**

**1. Abstract**

- The methods section should include the **study setting (hospital-based)** for clarity.
- It would strengthen the abstract if **effect size and confidence intervals** are reported along with P-values.
- Since the study is quasi-experimental, it would be appropriate to briefly **mention this as a limitation** in the abstract.

**2. Introduction**

- After discussing neonatal mortality, it would be helpful to include **data on maternal education levels in India**, as this directly relates to baseline knowledge of newborn care.
- The manuscript would benefit from a clearer discussion of **current challenges in the Indian context**, and how this educational intervention specifically addresses those barriers.

**3. Materials and Methods**

- The study duration was approximately **5 months**, yet the sample size is relatively small. This could be acknowledged as a limitation affecting generalizability.
- For reliability testing of the questionnaire, it is unclear whether this was conducted on **participants from the current study or a separate pilot group**. The number of participants involved in reliability testing should be specified.
- The rationale for selecting a **1-week interval between training sessions** is not explained and should be justified.
- Similarly, the reason for conducting the **post-test on the third day after childbirth** should be clarified.
- The researcher delivered the intervention and appears to have been involved in outcome assessment. If the assessor was aware of group allocation, this introduces a risk of **observer bias**, particularly for skill checklist scoring.

**Required action:**

 Clarify who conducted the skill assessments and whether assessors were blinded. If blinding was not performed, this should be explicitly stated and included as a limitation.

**4. Results**

- There appears to be a difference in **family income between the intervention and control groups**, with the control group having lower income levels. Since socioeconomic status can influence knowledge levels, this could introduce bias and should be acknowledged as a limitation.
- The manuscript states that there was **“**no missing data, **”** however, the participant flow diagram indicates dropouts prior to post-test assessment.

**Required action:**

 This statement should be revised to clarify that there were no missing data **among participants included in the final analysis**, rather than implying no attrition occurred.

**5. Discussion**

- It should be clearly stated whether there was a **statistically significant difference in knowledge between groups at the pre-test stage**, as this is important for interpreting the post-test results.

**Other Concerns (Data Consistency Issue)**

 In the breastfeeding domain, there is a discrepancy between the text and Table 5:

- The text reports that **9 participants (40%)** in the intervention group used a pillow under the newborn.
- However, Table 5 indicates that **16 participants (80%)** in the intervention group used a pillow.

For the control group, the text reports **7 participants (35%)**, which aligns with the table.

 This inconsistency should be clarified, and all reported data should be reviewed to ensure **consistency between text and tables**.

 The study addresses an important topic and presents potentially useful findings. However, improvements are needed in **methodological clarity, justification of study procedures, reporting transparency, and consistency of presented data**. Addressing these points will strengthen the overall quality and reliability of the manuscript.

**Final Recommendation**

**Minor to Moderate Revision**

Is the work clearly and accurately presented and does it cite the current literature?

Partly

If applicable, is the statistical analysis and its interpretation appropriate?

Yes

Are all the source data underlying the results available to ensure full reproducibility?

Yes

Is the study design appropriate and is the work technically sound?

Yes

Are the conclusions drawn adequately supported by the results?

Yes

Are sufficient details of methods and analysis provided to allow replication by others?

Yes

Reviewer Expertise:

Public Health and Epidemiology Research

I confirm that I have read this submission and believe that I have an appropriate level of expertise to confirm that it is of an acceptable scientific standard, however I have significant reservations, as outlined above.

# Reviewer report: Reviewer response for version 1

**DOI:** 10.5256/f1000research.196088.r479539

**Published:** 2026-05-04

**Authors:** Rakesh Kotha

**Affiliations:** 1 Osmania Medical College, Hyderabad, Telengana, India

The work is clearly and accurately presented and cites relevant current literature.

 The study design is appropriate (quasi-experimental with separate hospitals to avoid contamination) and technically sound.

 Sufficient methodological details are provided for replication.

 Statistical analysis (Mann-Whitney U test) and interpretation are appropriate for the non-normally distributed data.

 Source data are available on Zenodo (both underlying dataset and extended materials).

 Conclusions are well supported by the results.

 Detailed Comments

 Major Strengths

 The topic is highly relevant to neonatal health in LMICs and directly supports SDG 3.2.

 The use of simulation-based training is innovative and practical for resource-constrained settings.

 Results are impressive and clinically meaningful: post-test knowledge scores were markedly higher in the intervention group (mean 23.8 vs 9.25), and skill scores showed even larger differences (24.65 ± 1.56 vs 8.05 ± 2.35), with highly significant p-values (Z = -5.345 and Z = -5.144, both p < 0.001).

 Area-wise breakdown tables for thermoregulation, breastfeeding, and hygiene domains provide excellent granularity.

 Ethical clearances (IEC + CTRI registration) and data sharing statements are appropriately reported.

 Minor Suggestions 

 The non-randomized design is acceptable but could be more explicitly discussed as a limitation regarding potential selection bias.

 The variable time interval between the second training session and delivery (due to unpredictable labour) is acknowledged but could be quantified if data allow.

 A small number of references could be updated with more recent 2024–2025 newborn care guidelines if the authors wish, though the core content remains valid.

 Overall Scientific Soundness

 This is a well-executed, meaningful study with clear practical implications for community health nursing and maternal education programmes. No major revisions are required.

Is the work clearly and accurately presented and does it cite the current literature?

Yes

If applicable, is the statistical analysis and its interpretation appropriate?

Yes

Are all the source data underlying the results available to ensure full reproducibility?

Yes

Is the study design appropriate and is the work technically sound?

Yes

Are the conclusions drawn adequately supported by the results?

Yes

Are sufficient details of methods and analysis provided to allow replication by others?

Yes

Reviewer Expertise:

Neonatology

I confirm that I have read this submission and believe that I have an appropriate level of expertise to confirm that it is of an acceptable scientific standard.

# Reviewer report: Reviewer response for version 1

**DOI:** 10.5256/f1000research.196088.r476608

**Published:** 2026-04-27

**Authors:** Uppu Praveen

**Affiliations:** 1 All India Institute of Medical Sceince (AIIMS), Jammu, Jammu & Kashmir, India

This manuscript addresses an **important and highly relevant topic** in maternal and neonatal health, particularly in lower-middle-income settings. The focus on improving primipara mothers’ knowledge and skills through a **prenatal training module (PTM) combined with simulation** is both timely and practically valuable.

 The study demonstrates a **clear and meaningful contribution** to community health nursing and maternal education, highlighting the potential of structured training interventions to enhance newborn care practices and ultimately improve neonatal outcomes

**Relevance and Public Health Importance**

- The study targets a critical area— **newborn care and maternal preparedness**, which is directly linked to neonatal morbidity and mortality.
- The alignment with **Sustainable Development Goals (SDG 3)** strengthens its global significance.

**Innovative Intervention Approach**

- The use of a **simulation-based prenatal training module** is commendable.
- This approach enhances **practical learning and skill acquisition**, moving beyond traditional didactic teaching.

**Clear Study Objective and Outcomes**

- The research objectives are **well-defined and focused**.
- The use of both **knowledge and skill outcomes** provides a comprehensive evaluation of the intervention.

**Structured Methodology**

- The study follows a **systematic quasi-experimental design**.
- Ethical approval and clinical trial registration add to the **credibility and transparency** of the research

**Statistically Significant Findings**

- The results clearly demonstrate **significant improvement in both knowledge and skills**, supporting the effectiveness of the intervention.

**Practical Applicability**

- The intervention is **feasible, low-cost, and scalable**, making it highly suitable for implementation in resource-limited settings.
- The findings can inform **policy, nursing education, and community health programs**.

Overall, this is a **well-conducted and meaningful study** with strong practical implications, and the manuscript has the potential to make a **valuable contribution to the field of maternal and child health**.

Is the work clearly and accurately presented and does it cite the current literature?

Yes

If applicable, is the statistical analysis and its interpretation appropriate?

Yes

Are all the source data underlying the results available to ensure full reproducibility?

Yes

Is the study design appropriate and is the work technically sound?

Yes

Are the conclusions drawn adequately supported by the results?

Yes

Are sufficient details of methods and analysis provided to allow replication by others?

Yes

Reviewer Expertise:

Public Health, Community Health, Evidence Based Synthesis, Systematic reviews & Meta-analysis, RCT's, Biostatistics and Bioethics

I confirm that I have read this submission and believe that I have an appropriate level of expertise to confirm that it is of an acceptable scientific standard.
